# Experimental Investigation of Angular Stackgram Filtering for Noise Reduction of SPECT Projection Data: Study with Linear and Nonlinear Filters

**DOI:** 10.1155/2007/38516

**Published:** 2007-05-16

**Authors:** Antti P. Happonen, Matti O. Koskinen

**Affiliations:** ^1^Department of Clinical Physiology, Medical Imaging Center, Tampere University Hospital, P.O. Box 2000, 33521 Tampere, Finland; ^2^Institute of Signal Processing, Tampere University of Technology, P.O. Box 553, 33101 Tampere, Finland

## Abstract

We discuss data filtering prior to image reconstruction. For this kind of filtering, the radial direction of the sinogram is routinely employed. Recently, we have introduced an alternative approach to sinogram data processing, exploiting the angular information in a novel way. This new stackgram representation can be regarded as an intermediate form of the sinogram and image domains. In this experimental study, we compare the radial sinogram and angular stackgram filtering methods using physical SPECT phantoms. Our study is carried out by employing simple linear and nonlinear filters with ten different Gaussian kernels, in order to provide a comparable investigation. According to our results, angular stackgram filtering with the nonlinear filters provides the best resolution-noise tradeoff of the compared methods. Besides, stackgram filtering with these filters seems to preserve the resolution in an exceptional way. Visually, noise in the reconstructed images after stackgram filtering appears more “powdery” in comparison with radial sinogram filtering.

## 1. INTRODUCTION

In single photon emission computed tomography (SPECT), acquired planar projection images or scintigram data can be arranged as sinograms. A SPECT sinogram consists of radial intensity profiles of the tracer distribution in the body or object as a function of the projection angle. Images reconstructed from the acquired sinogram data represent two-dimensional (2D) transaxial slices of the three-dimensional (3D) radioactive distribution in the object. Since the image reconstruction process is an ill-posed problem, inherent noise in the projection data resulting from the random nature of radioactivity tends to degrade the quality of the reconstructed images. Numerous different methods for SPECT data filtering have been reported to alleviate the problem.

Statistical image reconstruction, such as the maximum-likelihood expectation-maximization (MLEM) algorithm [1], utilizing the probability distribution of the detected photon counts, provides a sophisticated way to deal with the noise problem. In MLEM reconstruction, however, noise and edge artifacts become visible after a large number of itera-tions. To avoid these unwanted artifacts, regularization methods such as early-stopping [2] or late stopping followed by postfiltering [3] are often used in practice. In the early-stopping method, the iterations are stopped before the full convergence in order to avoid “over fitting.” This introduces nonuniform resolution and contrast into the image. A large number of iterations followed by postfiltering is another regularization approach. After the late-stopping reconstruction, a proper modeling of the noise for filter design can be challenging due to the reconstruction artifacts.

**Figure 1:**
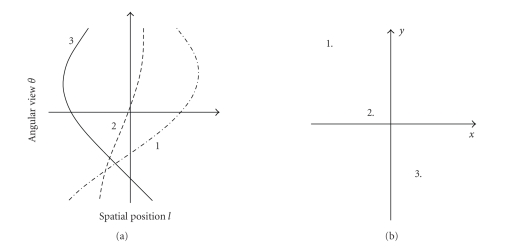
In (a), the sinogram is composed of sinusoidal signals. In SPECT, the sinusoids complete one cycle, that is, a full 360-degree view with different amplitudes and phases (the shown view corresponds to 180∘). The signals along the sinusoids contribute to the points or pixels in the reconstructed image, as shown in (b). The amplitude and phase of the sinusoidal signals (a) vary depending on the distance and spatial location of the points in the image (b).

A different approach to the quantum noise reduction is smoothing or filtering of the raw projection or sinogram data before image reconstruction. Then, in principle, simple and linear filtered back-projection (FBP) reconstruction can result in sufficient images, in terms of uniform resolution and contrast. Traditionally, the sinogram (see Figure 1) data are filtered only in the radial direction (i.e., along the projections). Well-known examples of radial data filtering are, for example, the Hanning and Butterworth lowpass filters [4], which are routinely employed in FBP reconstruction. In contrast, filtering along the angular direction of the data(i.e., across the projections of different angular views) is usually avoided since it introduces tangentially varying blurring into the reconstructed image [5].

**Figure 2:**
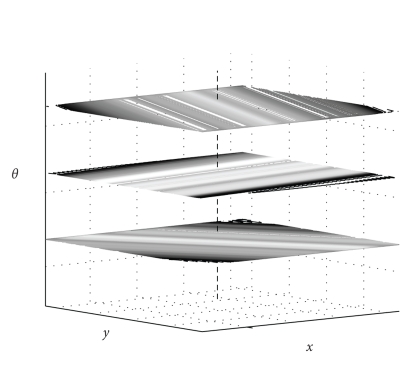
An illustration of a stackgram with three back-projected projections. The signals along the sinusoidal curves of the sinogram (as in Figure 1(a)) correspond to the signals through the layers of the stackgram. A signal such as this is illustrated with the dashed line in the shown stackgram; denoted as the locus signal. Notice the relation of the stackgram coordinates in comparison with the sinogram and image coordinates.

Recently, we have introduced an approach for sinogram data filtering that allows the use of the angular information of the projection data in a novel way. The proposed new approach is based on the stackgram domain [6, 7]. The stackgram representation of the sinogram data decomposes the signals along the sinusoidal trajectories of the sinogram into separate signals. These signals, denoted as locus-signals, can then be processed or filtered without affecting the other trajectory signals in the 3D stackgram (see Figure 2). In the 2D sinogram domain, in contrast, these signals intersect each other. The stackgram filtering technique offers a different and potential alternative to radial sinogram filtering [8, 9]. One-dimensional (1D) angular filtering in the stackgram domain does not introduce observable tangential distortion into the reconstructed images. Moreover, an interesting aspect of the behavior of the stackgram approach is that, in comparison with the radial sinogram filtering technique, it provides a more “powdery” or natural noise structure in the reconstructed images at a matched resolution. Our findings [8, 9] were obtained with *numerical phantom data* simulating acquisitions in positron emission tomography (PET), in which the imaging geometry differs from SPECT. Our previous studies [8, 9] gave experimental quantitative evaluations of the stackgram filtering technique by employing 20 different filters. In those studies [8, 9], we used ten different noise levels with a hundred noise realizations (for each level) to evaluate the noise reduction methods.

In this study, we compare the radial sinogram filtering technique with the angular stackgram approach with linear and nonlinear filters, similarly as in [8, 9]. In this experimental comparison, two *physical SPECT phantoms* were applied for quantitative and visual assessment of the two methods. We employed linear lowpass Gaussian filters and nonlinear L-filters. With these filters, the performances of the radial sinogram and the angular stackgram filtering techniques differ substantially [7–9]. The employed filters do not exploit any a priori knowledge nor provide optimal noise reduction of SPECT data. This investigation aims to give an insight into the performance of the novel stackgram approach and its possible tradeoffs in comparison with the commonly accepted radial sinogram filtering technique.

## 2. METHODS

### 2.1. Definition of stackgram

The Radon transform [10] maps a function *f(x, y)* to a sinogram *g(l, θ)*. The 3D stackgram (i.e., a stack of back-projections) is a decomposition of different curves consisting of the values along sinusoidal trajectories of the sinogram [6, 7]. The stack-operator *S* maps the sinogram *g(l, θ)* to a stackgram *h(x, y, θ)* as

(1) h (x,y,θ) =^Sg(l,θ) =g(x cos⁡θ+y sin ⁡θ,θ),

where *x* cos θ + y sin θ forms the sinusoidal curves on the sinogram for each (*x*, *y*) coordinate. The stack-operator simply reorganizes the sinogram values into the 3D domain. In some contexts, the 2D layers of the stackgram are referred to as ridge functions [11] (which are not associated with the 3D stackgram in [11]). The key function of the stackgram approach is to offer an environment to independently process each signal consisting of the values along the different sinusoidal trajectories. In the stackgram domain, these signals are referred to as locus-signals *h_x,y_*(θ) that can be written as

(2) $$h_{x,y}(\theta )=h(x,y,\theta ),\;\;\forall (x,y)\in \{x^{2}+y^{2}\leq C^{2}\}\subset {\mathbb{R}}^{2},$$

where *C* is a radius defining a support region and ℝ denotes the real set. The stack-operator *S*, as defined, is unbounded and the (*x, y*) layers of the stackgram are constant along each θ angle. Therefore, it is normally reasonable to bound the range of the stack-operator as {*h(x, y, θ)* = 0 |*x^2^* + *y^2^* >*C^2^* and θ ∉ [ 0 π)}. Alternatively, a so-called mollifier function [12] could be used for the bounding.

It is straightforward to show that the stack-operator is a linear operator. Let *g_1_* and *g_2_* be sinogram functions, and let *h_1_* and *h_2_* be the corresponding stackgrams. Thus, for each *a, b ∈ ℝ*,

(3) $$\begin{array}{l} S(ag_{1}+bg_{2})(l,\theta )\\ \;\;=(ag_{1}+bg_{2})(x\;\cos\;\theta +y\;\sin\;\theta ,\theta )\\ \;\;=ag_{1}(x\;\cos\;\theta +y\;\sin\;\theta ,\theta )+bg_{2}(x\;\cos\;\theta +y\;\sin\;\theta ,\theta )\\ \;\;=ah_{1}(x,y,\theta )+bh_{2}(x,y,\theta ). \end{array}$$

An inverse operator from the stackgram to the sinogram is not unique. This is due to the fact that the stackgram layers contain redundant information (see Figure 2). Thus, an inverse stack-operator *S* ^−1^ can be formulated by the simple relations *x* = *l* cos θ and *y* = *l*sin θ as [7]

(4) $$g(l,\theta )\;\hat{=}\;S^{-1}h=h(l\;\cos\;\theta ,l\;\sin\;\theta ,\theta ).$$

It can be verified that *g* = *S^−1^*(*Sg*).

The inversion described above, however, is not a feasible formulation for practical stackgram applications. In practice, an operator (such as filter) is employed to modify the locus-signals inside the support region (2). A more appropriate formulation, denoted as the generalized inverse stack-operator $S_{w}^{-1}$ [7], can be defined with both the Radon transform and a weight function *w*(*x, y; l, θ*) as

(5) g(l,θ)=^Sw−1h(x,y,θ)=∬w(x,y;l,θ)h(x,y,θ)δ(x⁢ cos θ+y sin θ−l)dx  dy,

where δ(⋅) is the Dirac delta function. Notice that the operator $S_{w}^{-1}$ is not a true mathematical inverse, since it projects a function from the 3D into the 2D domain, which cannot be a one-to-one mapping. The formulation (5) also gives an insight into our discrete implementation of the stackgram.

### 2.2. Implementation of stackgram

In the discrete case, we implemented the operators (1) and (5) as back- and reprojections by using 2D data rotations. For this, we employed the three-pass rotation algorithm, which decomposes 2D rotation (by angle θ) into three 1D translations as in [13]

(6) $$\begin{array}{ll} \text{rot}(\theta ) & =\left[\begin{array}{cc} \cos\theta & -\sin\theta \\ \sin\theta & \cos\theta \end{array}\right]\\ & =\left[\begin{array}{cc} 1 & -\tan\frac{\theta }{2}\\ 0 & 1 \end{array}\right]\cdot \left[\begin{array}{cc} 1 & 0\\ \sin\theta & 1 \end{array}\right]\cdot \left[\begin{array}{cc} 1 & -\tan\frac{\theta }{2}\\ 0 & 1 \end{array}\right] \end{array}$$

The 1D translations of (6) can be implemented by convolution of a sinc-function. A discrete sinc-interpolator [14] along with the three-pass rotation algorithm provides a reversible and high quality rotation procedure for 2D band-limited data.

Since the convolution corresponds to multiplication in the frequency or Fourier domain, a fast Fourier transform algorithm can be used in the implementation. Thus, in the discrete frequency domain, the sinc-interpolator with a translation parameter (*s* ∈ ℝ) for signals of odd number of samples (*N*) can be expressed as in [14]

(7) $$\text{​}\alpha^{s}(w)\text{​}=\{\begin{array}{ll} \exp(\frac{i2\pi sw}{N}) & \text{if}\;w=0,\ldots ,\frac{N-1}{2},\\ \exp(\frac{i2\pi s(w\text{​}-\text{​}N)}{N}) & \text{if}\;w\text{​}=\text{​}\frac{N\text{​}-\text{​}1}{2}\text{​}+\text{​}1,\ldots ,N\text{​}-\text{​}1. \end{array}$$

In the case of an even number of samples, the sinc-kernel of *w* with the parameter *s* can be formulated, respectively, as in [7]

(8) $$\beta^{s}(w)=\{\begin{array}{ll} \exp(\frac{i2\pi sw}{N}) & \text{if}\;w=0,\ldots ,\frac{N-1}{2}-1,\\ 1 & \text{if}\;w=\frac{N-1}{2},\\ \exp(\frac{i2\pi s(w-N)}{N}) & \text{if}\;w=\text{​}\frac{N\text{​}-\text{​}1}{2}\text{​}+\text{​}1,\ldots ,N-1. \end{array}$$

In this formulation (8), the highest frequency component at *w* = (*N* − 1) / 2 is treated differently compared to that in [14], in order to obtain a reversible kernel. The cost of this small difference is aliasing of the highest frequency component in data interpolation. By assuming that the data are properly sampled, however, we can accept this insignificant aliasing. It can be verified that α^s^(*w*) α^−*s*^ (*w*) = 1 and β^*s*^ (*w*)β^*−s*^(*w*) = 1.This means that a rotation of 2D data is reversible, when the three-pass algorithm (6) with the sinc-kernel (9) or (8) for the translations is employed. That is, fixed forward and back rotations of 2D data introduce negligible errors to the data in practice. These errors are mainly caused by the numerical accuracy of computer arithmetic.

Based on (6), (9), and (8), discrete stackgrams can be generated with simple linear operations as follows: (1) each sinogram projection is replicated over (*x, y*) -plane; (2) the resulting 2D data are rotated by appropriate angles; and (3) the rotated data are stacked as a stackgram. Steps 1–3 are analogous to (1). Respectively, the discrete stackgrams can be transformed back to sinogram data using the same procedure vice versa: the stackgram layers are back-rotated and then re-projected to 1D projections (this transformation would be analogous to (5) if the weight function *w* were chosen to be “averaging”). Our implementation ((6), (9), and (8)) enables a reversible sinogram-to-stackgram transformation, which is crucial in investigating the performance of stackgram filtering. This discrete transformation is linear. A precise description of our implementation can be found in [7].

### 2.3. Phantoms and data acquisition

We used two physical phantoms in our investigation: a hotspot phantom and the Hoffman brain phantom. The hotspot phantom is composed of two cylinders with diameters of 25 cm and 15 mm. The volume of the larger cylinder is 10 liters, while the smaller hotspot volume is 20 ml. The corresponding doses of Technetium-99 m for the water volumes were 36 MBq/l and 250 MBq/l, respectively. The phantom was acquired on a dual-head SPECT system (Siemens Nuclear Medicine) in tomographic mode. The time per projection was 20 s/step.

For the Hoffman phantom, a Tc-99 m dose of 120 MBq was employed. A dual-head SPECT system (Marconi Medical Systems) was operated in tomographic mode with an acquisition time of 45 s/step.

In both acquisitions, the full-rotation extent was 360 in 120 steps. The image format was 128 × 128. The specifications resulted in sinograms with size of 128 × 120. The stackgrams were generated over 360 ∘ view with 120 layers.

We employed the hotspot phantom data mainly for quantitative evaluation of the two filtering methods. Visual assessment of the techniques was carried out on the Hoffman phantom data.

### 2.4. Filters

Data filtering along the radial *l* -direction of the sinogram has a different effect on the data than stackgram filtering along the angular θ -direction. The angular θ -axis of the stackgram is orthogonal to the radial *l* -axis. A fixed filter provides neither a comparable evaluation of the resolution nor the noise reduction of the two methods. Thus, effects of the different filtering approaches need to be matched for a fair comparison by employing a range of filters.

In our investigation, we employed two different types of 1D filters: conventional linear Gaussian filters and nonlinear L-filters with Gaussian weights. The shift-invariant Gaussian filters were implemented as lowpass finite-impulse-response filters [15]. Each output point of the L-filter is obtained as a weighted sum of ordered data values in the moving window of length *N* as in [16]

(9) $$L\left(x;a\right)=\overset{N}{\underset{i=1}{\sum }}a_{i}x_{\left(i\right)},$$

where *a* is a weight vector and *x_(i)_* denotes ordered input data. We employed ten different Gaussian weights or kernels for both filter types, in order to match filtering effects (hereafter called “the Gaussian filters” and “the L-filters”). For the filter weights, we used samples from Gaussian distributions (μ = 0) with the standard deviations (σ) of 0.25, 0.5, 0.75, …, 2.5. The same weights were employed for both filter types (20 filters in total). The corresponding lengths of the filters were 3, 5, 7, …, 21 samples. The narrowest kernel corresponds to an identity filter in the case of Gaussian filters, whereas the same weights results in a median filter of length 3 in the case of L-filters (i.e., *a*≈[0, 1, 0] in (9)).

**Figure 3:**
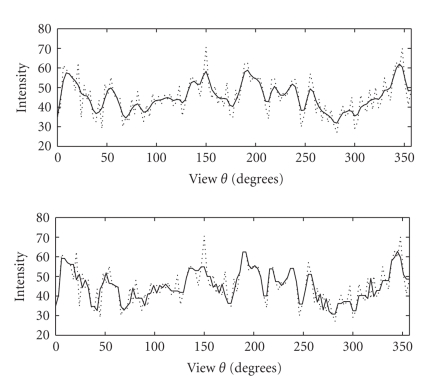
A selected locus-signal in the stackgram domain. The locus-signals contribute to the intensity values of the pixels in the reconstructed image. In the upper plot, the signal in the solid line was smoothed with a linear filter, whereas the same signal was processed with a nonlinear filter in the lower plot. The shown signal in the dashed line is the same initial noisy signal in both plots. The filtered signals illustrate the different performance of linear and nonlinear filters as well; nonlinear filters often preserve sharp transitions in signals better than linear filters.

In Figure 3, a selected locus-signal (2) before and after filtering is shown.

### 2.5. Evaluation methods

For evaluation of the filtering approaches, the acquired hotspot data were filtered employing the 20 filters in the two ways as follows: (1) along the radial sinogram direction, and (2) along the angular direction in the stackgram domain. This resulted in 40 projection data sets. The filtered data were reconstructed with the FBP algorithm using the ramp filter and a fixed attenuation correction (HERMES Nuclear Diagnostic). With the reconstructed data sets, we determined resolution-noise tradeoff curves for the two compared methods and for both filter types. This was accomplished by quantifying contrast recoveries (CRs) and coefficients of variation (CoVs) of the reconstructed FBP images. CoV versus CR plots provide a straightforward evaluation method for the resolution-noise tradeoff. The CR is defined as (H - B)/B. The symbols H and B represent average intensities of a high-count region (H) covering the “hotspot” in the data, and a background region (B) round the hotspot. The hot region H was a circle with a radius of two pixels, whereas the background B was a ring-shaped region with thickness of two pixels. In this way, the CR corresponds to a resolution measure. The CoV is defined as the standard deviation of the data over the mean of the data. After data filtering, the CoVs for the compared methods were quantified using a large uniform region in the reconstructed hotspot data.

Stackgram filtering tends to leave “powdery” or high frequency noise in the reconstructed images [7]. An evaluation method like the CoV favors images with non-high-frequency noise, such as that resulting from radial (lowpass) filtering of the sinogram data. Therefore, we also measured full-area-at-half-maximum (FAHM) values for the hotspot data without any noise measurements. The FAHM corresponds to full-width-at-half-maximum, but is a more convenient measurement in 2D data. The FAHMs were quantified for both filtering approach and for both filter types as follows: (1) a maximum count value of the hotspot was measured from the FBP reconstruction; (2) a cross-sectional area (in pixels) of the hotspot was determined at the half-maximum.

All the quantitative results (CR, CoV, and FAHM) were averaged over eight data values measured from eight similar transaxial slices of the FBP reconstructions of the hotspot data.

For the compared filtering techniques, the determined tradeoff curves (i.e., CoV versus CR) were also used to find comparable Gaussian filters as well as L-filters, in terms of noise reduction and resolution. These four matched filters were employed for the Hoffman phantom data. The filtered data aim to provide a comparable *visual assessment* of the two different filtering approaches with both filter types at the matched resolutions. The data were reconstructed with both the FBP (ramp filter) and MLEM algorithms without attenuation correction. In MLEM reconstruction (HERMES Nuclear Diagnostics), 100 iterations were used without additional filtering. Our assumption is that the MLEM algorithm (in addition to FBP) could utilize the stackgram-filtered sinograms due to the natural noise structure of the data [8, 9], resulting in pleasant and reliable images. In theory, data filtering prior to MLEM reconstruction would require additional data processing, such as NEC scaling [17], since the noise is presumably no longer Poisson-distributed. In this study, however, we did not apply such a preprocessing method.

## 3. RESULTS

### 3.1. Resolution-noise tradeoff

**Figure 4:**
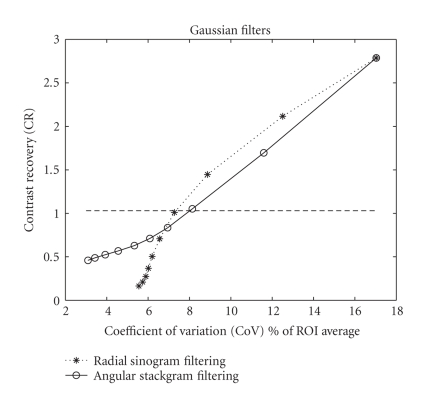
Resolution-noise tradeoff curves for the compared methods with the Gaussian filters. The points of the curves represent the 10 different filter kernels. The lines simply connect the points. The kernel width of the filters gets wider (i.e., the cut-off frequency of the lowpass filters decreases) from top-right to bottom-left. According to the shown curves, radial sinogram filtering provides a better tradeoff than angular stackgram filtering at the higher CR levels, whereas stackgram filtering performs better at the lower levels. The chosen or matched resolution level is also shown.

**Figure 5:**
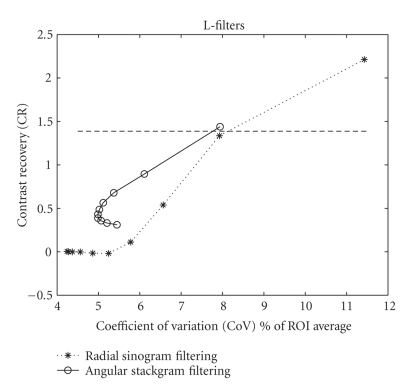
Resolution-noise tradeoff curves for the compared methods with the L-filters. The points of the curves represent the kernel widths of the 10 nonlinear filters. The lines simply connect the points. The kernel width gets wider (i.e., the filtering strength increases) from top-right to bottom. Angular stackgram filtering provides a better tradeoff than radial sinogram filtering for all the filter kernels. The chosen or matched resolution level is also shown.

Resolution-noise tradeoff curves of the compared methods are shown in Figures 4 and 5 for the Gaussian filters and the L-filters, respectively. With the linear Gaussian filters, radial sinogram filtering provides a better tradeoff than angular stackgram filtering at suitable resolution levels (see Figure 4). Overall, taking into consideration both filter types (Gaussian and L-filters), stackgram filtering with the L-filters provides the best tradeoff in terms of noise reduction at the appropriate resolution, although the differences in the tradeoffs are not so significant (compare Figure 4 to Figure 5). Note that in Figure 5 the measured data values for stackgram filtering below a CR of 0.5 are somewhat biased, since the curve suggests that noise or CoV increases. In this case, however, the filters below this CR value are insignificant in our investigation; because such filters are impractical for noise reduction (i.e., they provide a too narrow contrast).

### 3.2. Matched filters

In Figures 4 and 5, the chosen resolution levels are shown with dashed lines. In the case of the linear filters, the widths (σ) of the Gaussian filter kernels at the approximately-matched resolution are 1.0 (the standard Gaussian filter) and 0.75 for radial and stackgram filtering, respectively. Similarly, in the case of the L-filters, the chosen resolution level provides Gaussian filter weights with widths of 0.5 and 0.25 (median filter) for radial and stackgram filtering, respectively. Note that we do not try to match the linear and nonlinear filters, but the different filtering approaches (i.e., radial sinogram and angular stackgram) for the two filter types. These four different 1D filters were employed for the Hoffman data, to be discussed Section 3.4.

### 3.3. FAHM

**Figure 6:**
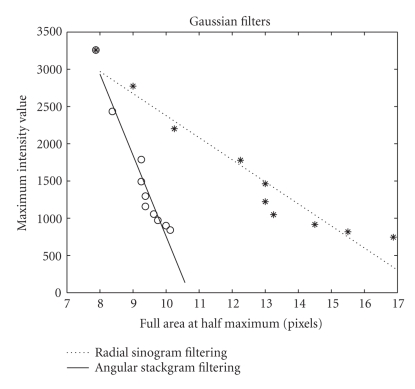
Maximum-intensity-value versus FAHM for the compared methods with the Gaussian lowpass filters. Polynomials of the first degree were fitted for the shown data points, which represent the 10 filter kernels. The cut-off frequency of the filters decreases from top-left to bottom-right. As can be seen angular stackgram filtering preserves the resolution (or FAHM) better than radial sinogram filtering, although the maximum-intensity-values or the maximum-counts decrease almost consistently.

**Figure 7:**
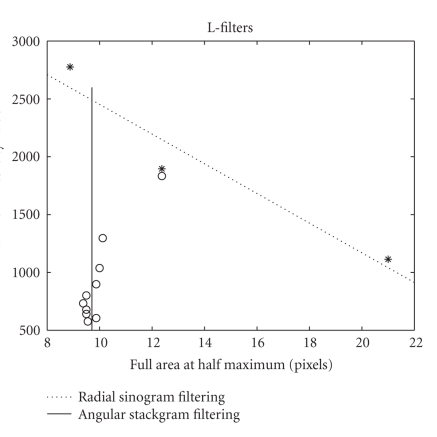
Maximum-intensity-value versus FAHM for the compared methods with the nonlinear L-filters. The data points represent the different filter kernels. The filter width or length increases from top to bottom. A polynomial of the first degree was fitted for the three radial filtering data points, whereas a line was fitted for the quantified stackgram data. Most of the obtained values for radial filtering were omitted from the plot and from the polynomial fit, since the values did not fit the appropriate scale. The highest stackgram data value was excluded from the line fit. Angular stackgram filtering seems to preserve the resolution (or FAHM) almost perfectly, at the cost of decreasing maximum count value.

FAHM versus maximum-intensity-value plots for the Gaussian filters are shown in Figure 6. As can be seen, stackgram filtering preserves the thickness (or FAHM) of the hotspot better than radial sinogram filtering, as the kernel width becomes wider. The L-filters, on the other hand, seem to preserve the FAHM in a quite exceptional way in stackgram filtering (see Figure 7). That is, regardless of the employed filter kernel, the thickness of the hotspot remains almost the same. In Figure 7, one point of the stackgram-filtered data seems to be apart from the rest of the quantified data values. A possible reason for this might be that the single data point represents a median filter, whereas the other points in the plot represent L-filters with a more Gaussian type of weights (9).

In both radial and stackgram filtering, the two matched L-filters (see Figure 5) perform rather equally also in terms of FAHM (see Figure 7). Thus, these L-filters should give practically as comparable a visual assessment of the two filtering methods as possible. On the other hand, in the case of Gaussian filters, the matched filters do not share (approximately) the same coordinates on the FAHM plot (see Figure 6). Again, the maximum-intensity-values predictably correspond to the measured CR values in the plot.

**Figure 8:**
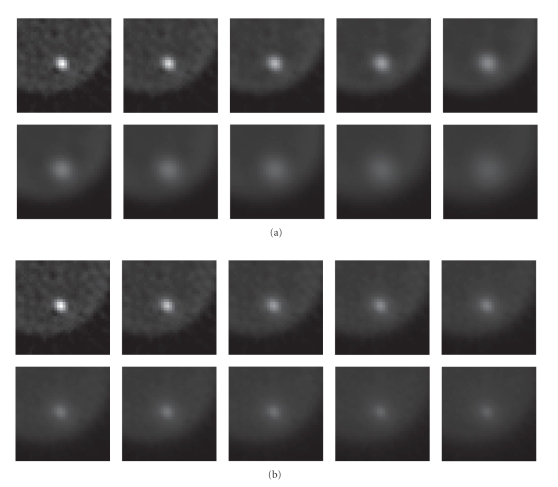
The hotspot data smoothed with the different Gaussian lowpass filters for the compared methods. In (a), cropped FBP images for radial sinogram filtering. In (b), the corresponding images for angular stackgram filtering. The filter kernel width of σ increases from left to right at each row in both figures.

**Figure 9:**
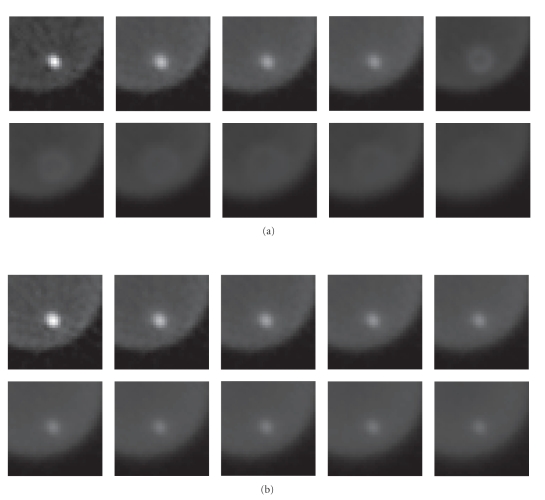
The hotspot data filtered with the different nonlinear L-filters for the compared methods. In (a), cropped FBP images for radial sinogram filtering, and the corresponding images for angular stackgram filtering in (b). The filter weights increase from left to right at each row in both figures. In (a), notice that after the second image (top row), the peak has a totally different shape compared to the initial shape. In (b), the width of the peak in the images remains almost invariable regardless of the filter width.

A transaxial slice of the filtered hotspot data is shown in Figure 8 (Gaussian filtering) and Figure 9 (L-filtering). Figure 9(a) illustrates what sort of geometrical distortion the nonlinear L-filters, in the case of radial sinogram filtering, can introduce into the reconstructed images (the ring-shaped hotspots). A similar effect cannot be observed after stackgram filtering (see Figure 9(b)). Figure 8 and 9 aim to support the shown curves (Figures Figures 4–7) visually.

### 3.4. Hoffman data

**Figure 10:**
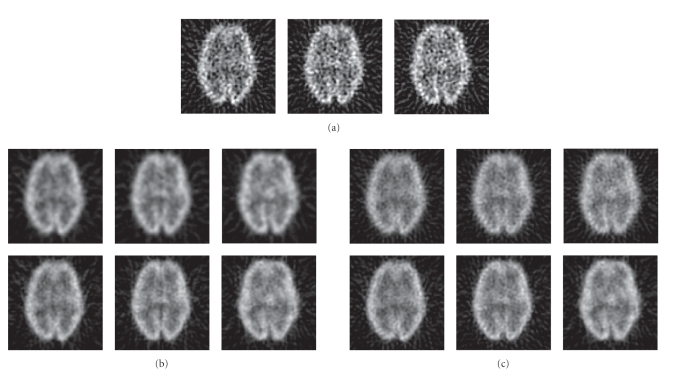
Three transaxial slices of the physical Hoffman phantom. In (a), FBP-reconstructed images without any noise reduction. In (b), radial sinogram filtering: FBP images for Gaussian filtering (top row) and for L-filtering (bottom row) at the matched resolutions (see Figures 4 and 5). In (c), angular stackgram filtering: FBP images for Gaussian filtering (top row) and for L-filtering (bottom row) at the matched resolutions (Figures 4 and 5). The noise variation seems to be higher after angular stackgram filtering (c) in comparison with radial sinogram filtering (b). All images share a common grayscale.

Figure 10(a) shows FBP images of the Hoffman phantom (FBP with ramp filter). In Figures 10(b) and 10(c) (top row), FBP images of the Hoffman data are shown for radial sinogram and angular stackgram filtering with the matched Gaussian filters. These FBP images are congruent with the shown curves (Figures 4 and 6); stackgram filtering tends to leave more noise variation in the images (or this can be regarded as a powdery noise structure).

Figures 10(b) and 10(c) (bottom row) show FBP images for radial and stackgram filtering with the matched L-filters. As expected (Figures 5 and Figure 7), the images of the different filtering methods appear rather similar. There are, however, observable differences in the images, especially in the structure of noise. This effect (as in the case of Gaussian filters) can be explained by the fact that stackgram filtering preserves the sinusoidal structure of the filtered sinograms, unlike radial sinogram filtering.

**Figure 11:**
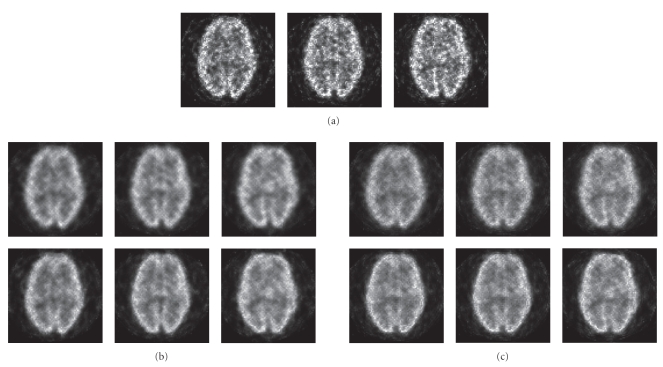
In (a), three (late-stopping) MLEM-reconstructed transaxial slices of the Hoffman phantom. In (b), radial sinogram filtering: MLEM images for Gaussian filtering (top row) and for L-filtering (bottom row) at the matched resolutions (see Figures 4 and 5). In (c), angular stackgram filtering: MLEM images for Gaussian filtering (top row) and for L-filtering (bottom row) at the matched resolutions (Figures 4 and 5). All images share a common grayscale.

As stated, the MLEM algorithm could perhaps utilize the stackgram-filtered data due to the “powdery” noise structure resulting in visually pleasing images without annoying reconstruction artifacts, although the noise after filtering would not be exactly Poisson-distributed. Figure 11(a) shows late-stopping MLEM reconstructions of the Hoffman data (100 iterations). In Figures 11(b) and 11(c) (top row), MLEM images of 100 iterations for radial sinogram and angular stackgram filtering with the matched Gaussian filters are shown. Figures 11(b) and 11(c) (bottom row) show the same transaxial slices after filtering with the matched L-filters. As can be observed the MLEM images (Figures 11(b) and 11(c)) do not differ substantially. However, the noise structure in the images after linear stackgram filtering (see Figure 11(c), top row) appears to be most similar to that in the late-stopping MLEM reconstruction (see Figure 11(a)), as compared to the rest of the images (see Figure 11).

## 4. DISCUSSION

The results of this investigation are congruent with our previous experience of stackgram filtering in numerical PET phantom data (192 × 192 resolution with 256 views) [8, 9]. There are, however, some minor differences resulting (perhaps) from the different imaging geometry (360°) and resolution (128 × 128) of SPECT. First, as regards the resolution-noise tradeoffs (Figures 4 and 5), stackgram filtering seems to provide quantitatively better results with narrower filter kernels than does radial sinogram filtering at the appropriate resolution levels. Previously, we have found the opposite. Secondly, the differences in the noise after radial and stackgram filtering of the SPECT data (see Figure 10) seem not to be as evident as we have found with PET data [8, 9].

Stackgram filtering performs better with the nonlinear L-filters than with the linear Gaussian filters (Figures 4 and 5). We believe that this kind of effect holds more generally for linear and nonlinear type of filters in 1D stackgram filtering. This can be explained by the fact that the stackgram (1) contains redundant information, that is, each layer of the stackgram is constant at angle θ. This means that several locus-signals to be filtered share the same data points, forming, for example, noise peaks. Generally, nonlinear filters remove this kind of redundancy or correlation more accurately than linear filters.

Stackgram filtering seems to preserve the resolution or FAHM in a quite exceptional way as the kernel width increases, whereas radial sinogram filtering performs conventionally (Figures 6 and 7). The following heuristic reasoning aims to give an insight into this effect. Theoretical considerations would naturally provide a more solid explanation, but these are outside the scope of our investigation. As noticed, stackgram filtering “shrinks” the intensity scale of the data when the filter widths get wider, but do not cause spatial blurring in the same way as radial filtering of the sinogram. This can be understood by the structure of the stackgram, which is like a stack of back-projections. Summing up the stackgram along the θ -axis results in a back-projected (BP) image, which can be regarded as a blurred counterpart of the reconstructed image. The BP image has a similar shrunken intensity scale as the stackgram filtered data with the wide filters (Figures 6 and 7). Heuristically, if the stackgram data are filtered with more and more “powerful” or averaging 1D filters (which would be inappropriate for noise reduction), then each layer of the stackgram converges to a back-projected image, that is, the average of all the layers. This would result in data similar to those indicated with the wide filter widths (Figures 6 and 7).

The stackgram allows us to exploit the angular direction of the projection data without introducing tangential blurring to the reconstructed image, as can be observed, for example, in Figures 8 and 9 (i.e., the shape of the hotspot remains circular). In contrast, filtering of the sinogram data along the angular direction introduces observable nonuniform and tangential spatial blurring to the reconstructions [5]. The tangential distortion is extremely obvious in the case of 1D filters. To avoid this undesirable effect, some techniques for sinogram domain filtering utilizing the angular direction have been published [18, 19]. These techniques, however, often restrict the available filters or require adjustments to them. In angular stackgram filtering, any 1D filter or denoising technique can be employed for the noise reduction in locus-signal (2) filtering without the need for regulation of tangential blurring. This claim or observation is based on our experience in stackgram filtering on conventional emission tomography data [7]. Stackgram filtering, on the other hand, requires more computational time and especially computer memory space, as compared to sinogram-domain-based filtering methods.

In our resolution-noise evaluations, we have explicitly assumed that the two filtering approaches led to images with spatially uniform resolution if shift invariant filters were employed. In radial sinogram filtering, the assumption of uniform resolution is validated by the central slice theorem [10]. Based on our experimental studies [8], stackgram filtering with linear shift-invariant filters also introduces a spatially uniform resolution and contrast to the reconstructed images (note that the applied discrete stackgram transformation is linear). Thus, the resolution-noise tradeoffs, as well as the FAHM curves for the linear Gaussian filters (Figures 4 and 6), should describe the performance of the compared filtering approaches reliably and predictably. On the other hand, the employed nonlinear L-filters (9) are based on order statistics, in which case a filtering effect on an isolated impulse (or on a larger “hotspot”) is not predictive for more general structures. In Figure 9(a), this explains the distorted shape of the hotspot after radial filtering with the wide filters. However, the plots (Figures 5 and 7) seem to provide appropriate evaluations of the filtering approaches, and highlight the different performances.

In this study, our objective was to compare the novel stackgram approach with the conventional radial sinogram filtering technique, not to develop optimal stackgram-based filters. To develop suitable filters for SPECT, it would be necessary to study further the noise properties of the SPECT stackgrams. It is quite obvious, however, that the noise distribution of the locus-signals (2) in the stackgram domain (1) follows a Poisson distribution, since the SPECT sinogram data are simply reorganized in the stackgram. Furthermore, in general, a sufficiently large subset of data presumably follows the same distribution as the initial data set (cf. a sinusoidal trajectory signal as the subset and the sinogram as the complete set). Besides, in our previous studies, we have found that the noise distribution of the locus-signals in discrete PET stackgrams follows approximately a generalized Poisson distribution [20]. It is important to note that the implementation of the discrete stackgrams ((6) and (7) or (8)) implicitly introduces minor changes to the noise in the locus-signals, for example, due to the ringing effect associated with sinc-interpolation. It is also worth noticing that many of the locus-signals to be filtered contain rather a constant intensity range (such as in Figure 3). This implies that the (Poisson) noise has a constant variation in these 1D signals, that is, the noise distribution is actually close to Gaussian. In summary, considering the discussion above, the applied nonlinear L-filters with Gaussian weights (9) may turn out to be close to an optimal filter design for noise reduction of SPECT stackgrams.

Statistical iterative reconstruction algorithms can take into account the Poisson nature of noise in the emission projection data. The iterative methods such as MLEM are therefore routinely used nowadays in image reconstruction. It is a well-known fact that radial filtering of the SPECT projections modifies the Poisson distribution of the noise, especially with lowpass filters, thus preventing the use of the MLEM algorithm in theory. A similar modification effect on the noise distribution introduced by stackgram filtering is not so obvious, because the angular direction of the stackgram is orthogonal to the radial direction or the acquisition plane. This supposition, and our previous studies [8, 9], gave us the motivation to test the stackgram-filtered data using the MLEM reconstruction algorithm, although the noise distribution of the filtered data is unknown and would require further studies. However, since the discrete stackgram transformation is linear, we assumed that (particularly) a linear filter could approximately preserve the Poisson noise distribution in stackgram-filtered sinograms. Thus, the use of the MLEM algorithm would lead to visually pleasing or artifact-free images, due to lower noise variation in the sinogram data. Stackgram filtering, however, does not seem to provide significant differences in the reconstructed MLEM images, as compared to the “unnatural” radial-filtered data (see Figure 11). Nevertheless, the stackgram approach incorporated into the MLEM algorithm could be a useful, or at least interesting, future direction of research. Other authors have reported preliminary results of a similar approach to image reconstruction in [21].

## 5. CONCLUSION

For noise reduction of SPECT sinograms, angular stackgram domain filtering offers an alternative to conventional radial sinogram domain filtering. We compared these two filtering approaches with both linear Gaussian filters and nonlinear L-filters using physical SPECT phantoms. The chosen filters were mainly used for evaluation purposes of the two filtering domains. In this experimental investigation, stackgram filtering with the nonlinear L-filters provides the best resolution-noise tradeoff. At appropriate resolution levels, however, the differences in the resolution-noise tradeoffs of the compared filtering approaches seem to be quite small.

The overall performance of 1D stackgram filtering differs significantly from 1D radial sinogram filtering. That is, angular filtering in the stackgram domain seems not to introduce conventional blurring to the reconstructed images, but rather shrinks the intensity scale of the data. Furthermore, the noise in the images after stackgram filtering does not appear to be as smooth as in the case of radial filtering at the same resolution.

Our investigation shows the potential of stackgram do-main filtering. More (theoretical) studies, however, are need-ed to explore the novel stackgram approach in order to develop suitable or optimal stackgram-based filtering techniques for raw SPECT data.
